# Datasets for residential GSHP analysis by climate in the United States

**DOI:** 10.1016/j.dib.2020.106523

**Published:** 2020-11-14

**Authors:** Rebecca Neves, Heejin Cho, Jian Zhang

**Affiliations:** a Department of Mechanical Engineering, Mississippi State University, P.O. Box 9552, Mississippi State, MS 39762, USA; b Richard J. Resch School of Engineering, University of Wisconsin-Green Bay, Green Bay, WI 54311, USA

**Keywords:** Geothermal, Residential, United States, EnergyPlus™ Heat pump coefficient generator, Water-to-air heat pump, Ground source

## Abstract

This data captures climate information and HVAC energy use for a baseline prototype home and for a replacement alternative energy home. The baseline home is a traditional DX cooling/gas furnace system, and the alternate system is a geothermal heat pump. Cooling degree days (CDD), heating degree days (HDD) and relative humidity were gathered from historical weather data for 12 cities across the contiguous United States [1], [2]. Geothermal heat pump coefficients were generated as inputs to EnergyPlus™ simulation software. These heat pump coefficients are generated by compiling heat pump performance data from 5 market leading, high efficiency residential geothermal heat pump manufacturers. These coefficients can be used to represent a general, market available heat pump in 2-ton, 3-ton, and 4-ton capacities. Baseline prototype home energy use by city was generated by EnergyPlus™ using the prototype home download file from www.energy.gov and the respective weather file for that city. This data can be interpreted as energy use per month by certain HVAC components. The ground source heat pump (GSHP) home energy use by city was generated from EnergyPlus™ and the respective city weather file. The GSHP model was created by the authors to model the alternate closed loop, GSHP system. Reuse potential for heat pump coefficients and home energy use analysis is strong.

## Specifications Table

**Table:** 

Subject	Renewable Energy, Sustainability and the Environment
Specific subject area	Geothermal heat pump energy efficiency compared to traditional HVAC system in residential buildings in the United States. Financial incentives and analysis by climate.
Type of data	Table
How data were acquired	EnergyPlus™ Simulation Tool version 9.2.0
Data format	Raw Analyzed (Heat Pump Coefficients)
Parameters for data collection	Monthly energy use by city, raw data measured in Joules [J] by HVAC system component
Description of data collection	Energy use for baseline and GSHP model data was collected using EnergyPlus™ simulation tool; EnergyPlus™ heat pump coefficient data was collected using the Water-To-Air Heat Pump Coefficient Generator
Data source location	All data was collected by simulation in the following locations: Miami, Florida Phoenix, Arizona Memphis, Tennessee Las Vegas, Nevada Los Angeles, California Baltimore, Maryland Portland, Oregon Des Moines, Iowa Reno/Tahoe, Nevada Helena, Montana Duluth, Minnesota Gunnison, Colorado
Data accessibility	Repository name: Mendeley Data Data identification number: [provide number] Direct URL to data: doi:10.17632/xnbwy8s2gy.1
Related research article	Rebecca Neves, Heejin Cho, Jian Zhang, “State of the Nation: Customizing Energy and Finances for Geothermal Technology in the United States Residential Sector,” *Renewable and Sustainable Energy Review*, 2020, 110463, https://doi.org/10.1016/j.rser.2020.110463.

## Value of the Data

• The data are useful because it quantifies energy use for two HVAC systems for comparison. The cities chosen represent 12 diverse climate zones across the United States. The GSHP model built in EnergyPlus™ is a template that can be applied to any location. The heat pump coefficient data are particularly useful because they represent an overall performance curve across several high-efficiency geothermal heat pump manufacturers. Individual manufacturer's data and heat pump coefficients resulted in enough variability to desire a general, or normalized, heat pump performance curve for this simulation. Because the study performed was not comparing one manufacturer to another, the heat pump performance needed to represent the market-available equipment. The data presented will save much time and effort for future researchers looking for general heat pump performance coefficients for EnergyPlus™ input. Capacities represented are 2, 3 and 4-ton systems.
• The data presented benefits researchers studying geothermal heat pump performance, particularly for residential applications. The EnergyPlus™ heat pump coefficients benefit those seeking performance curves for an overall market-available geothermal heat pump, not tied to a specific manufacturer. The energy use data is useful to researchers seeking to quantify usage and financial savings for alternative energy HVAC systems. The original, or baseline, system is a DX cooling / natural gas heating traditional system. The replacement system is a ground source heat pump that has been sized accounting for soil characteristics local to specific regions of the United States.
• How can these data be used for further insights and development of experiments? Further insights and development of experiments can benefit from the data presented. The heat pump performance coefficients apply for any analysis of a residential geothermal heat pump that is simulated with EnergyPlus™. The data can be used as direct inputs into the Water-To-Air Heat Pump cooling coil and heating coil objects. The home energy use data is useful for further insights to compare or contrast with other cities across the country or the globe. This comparison is possible with the baseline prototype home data or the geothermal heat pump system data.

## 1 Data Description

Dataset contains four folders grouping the data appropriately and described below.

The folder titled “Baseline Prototype Home Energy Use by City” contains 1 file.

• The file “All Cities System Comparison.xlsx” contains month by month energy use for the HVAC system operation for all 12 cities in the study.

The folder titled “CDD, HDD and RH by City” contains 1 file [1], [2].

• The file “12 Cities Temperature Humidity.xlsx” contains a summation by month of the heating degree days, cooling degree days, and average relative humidity for all 12 cities in the study.

The folder titled “Geothermal Heat Pump Coefficient Generator” contains 6 files.

• “WaterAir_PE_Cooling 024 - Combined” contains the heat pump performance data for five manufacturers and the resulting EnergyPlus™ heat pump coefficients for a 2-ton heat pump cooling coil.
• “WaterAir_PE_Cooling 036 - Combined” contains the heat pump performance data for five manufacturers and the resulting EnergyPlus™ heat pump coefficients for a 3-ton heat pump cooling coil.
• “WaterAir_PE_Cooling 048 - Combined” contains the heat pump performance data for five manufacturers and the resulting EnergyPlus™ heat pump coefficients for a 4-ton heat pump cooling coil.
• “WaterAir_PE_Heating 024 - Combined” contains the heat pump performance data for five manufacturers and the resulting EnergyPlus™ heat pump coefficients for a 2-ton heat pump heating coil.
• “WaterAir_PE_Heating 036 - Combined” contains the heat pump performance data for five manufacturers and the resulting EnergyPlus™ heat pump coefficients for a 3-ton heat pump heating coil.
• “WaterAir_PE_Heating 048 - Combined” contains the heat pump performance data for five manufacturers and the resulting EnergyPlus™ heat pump coefficients for a 4-ton heat pump heating coil.

The folder titled “GSHP Home Energy Use by City” contains 24 files.

• The file “SF_Arizona_Phoenix-Sky.Harbor.Intl.AP.722780_Composite HP036Meter.csv” contains the electric meter readings by month for the 3-ton system broken down into the components that make up the HVAC system for Phoenix, AZ.
• The file “SF_Arizona_Phoenix-Sky.Harbor.Intl.AP.722780_Composite HP036Table.csv” contains the comprehensive tabular view of climactic and end use component details for Phoenix, AZ.
• The file “SF_California_Los.Angeles.Intl.AP.722950 Geothermal Composite HP024Meter.csv” contains the electric meter readings by month for the 2-ton system broken down into the components that make up the HVAC system for Los Angeles, CA.
• The file “SF_California_Los.Angeles.Intl.AP.722950 Geothermal Composite HP024Table.csv” contains the comprehensive tabular view of climactic and end use component details for Los Angeles, CA.
• The file “SF_Colorado_Gunnison.County.AWOS.724677_Geothermal HP024Meter.csv” contains the electric meter readings by month for the 2-ton system broken down into the components that make up the HVAC system for Gunnison, CO.
• The file “SF_Colorado_Gunnison.County.AWOS.724677_Geothermal HP024Table.csv” contains the comprehensive tabular view of climactic and end use component details for Gunnison, CO.
• The file “SF_Florida_Miami.Intl.AP.722020_Geothermal Composite HP036Meter.csv” contains the electric meter readings by month for the 3-ton system broken down into the components that make up the HVAC system for Miami, FL.
• The file “SF_Florida_Miami.Intl.AP.722020_Geothermal Composite HP036Table.csv” contains the comprehensive tabular view of climactic and end use component details for Miami, FL.
• The file “SF_Iowa_Des.Moines.Intl.AP.725460_gasfurnace_crawlspace_Geothermal Composite HP036Meter.csv” contains the electric meter readings by month for the 3-ton system broken down into the components that make up the HVAC system for Des Moines, IA.
• The file “SF_Iowa_Des.Moines.Intl.AP.725460_gasfurnace_crawlspace_Geothermal Composite HP036Table.csv” contains the comprehensive tabular view of climactic and end use component details for Des Moines, IA.
• The file “SF_Maryland_Baltimore-Washington.Intl.AP.724060 Geothermal Composite HP036Meter.csv” contains the electric meter readings by month for the 3-ton system broken down into the components that make up the HVAC system for Baltimore, MD.
• The file “SF_Maryland_Baltimore-Washington.Intl.AP.724060 Geothermal Composite HP036Table.csv” contains the comprehensive tabular view of climactic and end use component details for Baltimore, MD.
• The file “SF_Minnesota_Duluth.Intl.AP.727450 Geothermal Composite HP036Meter.csv” contains the electric meter readings by month for the 3-ton system broken down into the components that make up the HVAC system for Duluth, MN.
• The file “SF_Minnesota_Duluth.Intl.AP.727450 Geothermal Composite HP036Table.csv” contains the comprehensive tabular view of climactic and end use component details for Duluth, MN.
• The file “SF_Montana_Helena.Rgnl.AP.727720_gasfurnace_crawlspace_Geothermal Composite HP036Meter.csv” contains the electric meter readings by month for the 3-ton system broken down into the components that make up the HVAC system for Helena, MT.
• The file “SF_Montana_Helena.Rgnl.AP.727720_gasfurnace_crawlspace_Geothermal Composite HP036Table.csv” contains the comprehensive tabular view of climactic and end use component details for Helena, MT.
• The file “SF_Nevada_Las.Vegas-McCarran.Intl.AP.723860 Geothermal Composite HP036Meter.csv” contains the electric meter readings by month for the 3-ton system broken down into the components that make up the HVAC system for Las Vegas, NV.
• The file “SF_Nevada_Las.Vegas-McCarran.Intl.AP.723860 Geothermal Composite HP036Table.csv” contains the comprehensive tabular view of climactic and end use component details for Las Vegas, NV.
• The file “SF_Nevada_Reno-Tahoe.Intl.AP.724880_gasfurnace_crawlspace_Geothermal Composite HP036Meter.csv” contains the electric meter readings by month for the 3-ton system broken down into the components that make up the HVAC system for Reno, NV.
• The file “SF_Nevada_Reno-Tahoe.Intl.AP.724880_gasfurnace_crawlspace_Geothermal Composite HP036Table.csv” contains the comprehensive tabular view of climactic and end use component details for Reno, NV.
• The file “SF_Oregon_Portland.Intl.AO.726980_Geothermal Composite HP036Meter.csv” contains the electric meter readings by month for the 3-ton system broken down into the components that make up the HVAC system for Portland, OR.
• The file “SF_Oregon_Portland.Intl.AO.726980_Geothermal Composite HP036Table.csv” contains the comprehensive tabular view of climactic and end use component details for Portland, OR.
• The file “SF_Tennesse_Memphis.Intl.AP.723340 Geothermal Crawlspace Composite HP036Meter.csv” contains the electric meter readings by month for the 3-ton system broken down into the components that make up the HVAC system for Memphis, TN.
• The file “SF_Tennesse_Memphis.Intl.AP.723340 Geothermal Crawlspace Composite HP036Table.csv” contains the comprehensive tabular view of climactic and end use component details for Memphis, TN.

## 2 Experimental Design, Materials and Methods

Experimental design was performed by modelling a geothermal heat pump HVAC system for a prototype home file. Materials included EnergyPlus™ simulation tool and Microsoft Excel. Baseline energy use data was acquired through simulation of the prototype home file for each city. The baseline home consisted of a traditional DX cooling system and natural gas furnace heating system. The retrofit geothermal system required several inputs including the heat pump cooling and heating coil coefficients, ground heat exchanger parameters, soil conductivity and specific heat capacity, and borefield type. For the heat pump coefficient data, an extensive amount of data entry was required from the engineering specifications published by various heat pump manufacturers. Once reported, the annual energy use from the baseline system is compared to the annual energy use from the retrofit geothermal heat pump system. This comparison allowed for a city-specific financial analysis to determine the viability of geothermal system implementation. Methods were simulation executions. Detailed information on the methods is provided in Ref. [3].

## CRediT Statement

Rebecca Neves: Conceptualization, Methodology, Software, Formal Analysis, Writing – Original Draft, Visualization Heejin Cho: Writing – Review & Editing, Supervision. Jian Zhang: Writing – Review & Editing.

## Declaration of Competing Interest

The authors declare that they have no known competing financial interests or personal relationships which have, or could be perceived to have, influenced the work reported in this article.

## Appendix Supplementary materials

Image, application 1
